# Elevated hepcidin serum level in response to inflammatory and iron signals in exercising athletes is independent of moderate supplementation with vitamin C and E

**DOI:** 10.14814/phy2.12475

**Published:** 2015-08-04

**Authors:** Víctor Díaz, Ana B Peinado, Laura Barba-Moreno, Sandro Altamura, Javier Butragueño, Marcela González-Gross, Birgit Alteheld, Peter Stehle, Augusto G Zapico, Martina U Muckenthaler, Max Gassmann

**Affiliations:** 1 Institute of Veterinary Physiology, Vetsuisse Faculty, and Zurich Center for Integrative Human Physiology (ZIHP), University of ZurichZurich, Switzerland; 2 Department of Health and Human Performance, Technical University of MadridMadrid, Spain; 3 Department of Pediatric Oncology, Hematology and Immunology, Molecular Medicine Partnership Unit, University of HeidelbergHeidelberg, Germany; 4 Institute of Nutritional and Food Sciences. Nutritional Physiology, Rheinische Friedrich Wilhelms UniversitätBonn, Germany; 5 Department of Physical Education, Complutense University of MadridMadrid, Spain; 6 Universidad Peruana Cayetano Heredia (UPCH)Lima, Peru

**Keywords:** Endurance, interleukin-6, iron deficiency, performance, vitamins

## Abstract

Iron deficiency among endurance athletes is of major concern for coaches, physicians, and nutritionists. Recently, it has been observed that hepcidin, the master regulator of iron metabolism, was upregulated after exercise and was found to be related to interleukin-6 (IL-6) elevation. In this study performed on noniron deficient and well-trained runners, we observed that hepcidin concentrations remain elevated in response to inflammatory and iron signals despite a 28-days supplementation period with vitamins C (500 mg/day) and E (400 IU/day).

## Introduction

Iron is an essential element for adequate delivery of oxygen to tissues and is an indispensable component of oxygen storage and transport proteins such as hemoglobin (Hb), myoglobin, or cytochromes involved in mitochondrial respiration (Burratti et al. 2014). The hepatic antimicrobial peptide hepcidin is the master regulator of iron homeostasis and modulates duodenal iron absorption and iron recycling in macrophages (for review see reference (Steinbicker and Muckenthaler 2013). Iron deficiency or an inadequate iron status may both ultimately reduce physical performance and may negatively affect the immune system and neuronal functioning (Beard and Tobin 2000). Athletes are frequently diagnosed with inadequate iron levels, particularly those involved in endurance sports (Schumacher et al. 2002; Suedekum and Dimeff 2005; Milic et al. 2011; Hinton 2014). Therefore, the prevention of iron deficiency, especially in endurance athletes, is of major importance for physicians and coaches as regular training practices, in occasions accompanied by inadequate iron intake, may cause iron deficiency and/or anemia through intravascular hemolysis, hematuria, gastrointestinal bleeding, sweating, or disturbance of iron homeostasis (McInnis et al. 1998; Babic et al. 2001; DeRuisseau et al. 2002).

Exercise is known to increase inflammation markers, especially that of interleukin-6 (IL-6) that activates hepcidin expression (Pedersen et al. 2001; Peeling et al. 2008, 2009a), even in mountaineers upon ascent (Goetze et al. 2013; Altamura et al. 2015). In turn, elevated hepcidin levels reduce dietary iron absorption and release from macrophages that recycle iron from damaged erythrocytes and thus contribute to reduce iron availability in serum (Steinbicker and Muckenthaler 2013; Gassmann and Muckenthaler 2015). Exercise-induced IL-6 elevation can be blocked upon a 28-days supplementation period with vitamin C and E (Fischer et al. 2004). In this study, we aimed to investigate the relative contribution of inflammation and increased circulating levels of iron. We hypothesized that the hepcidin response should be blunted after supplementation with vitamins C and E, both vitamins being expected to reduce the postexercise inflammatory response that triggers hepcidin expression.

## Material and Methods

### Participants

Ten well-trained and nonsmoking male subjects (triathletes and marathon runners who train 5–6 sessions a week) studying Sport Sciences at the Technical University in Madrid (Spain) volunteered to participate in this study (26.9 ± 6.7 years, 69.3 ± 8.8 kg and 176.6 ± 7.5 cm, with maximum oxygen consumption 69.8 ± 5.7 mL/min/kg). Individuals were selected after a first screening to recruit solely subjects without iron deficiency (i.e., serum ferritin >50 *μ*g/L, [Hb] >115 g/L and transferrin saturation >16%) (Peeling et al. 2008; Peeling 2010) and who were not consuming dietary supplements. The study was approved by the ethical committee of the Technical University of Madrid and an informed consent was obtained prior the experiments.

### Experimental design

The volunteers performed a first incremental test, and 1 week later the first test without any vitamin supplementation. The same day started receiving an oral supplementation with a combination of ascorbic acid (Vit C, 500 mg/day) and RRR-*α*-tocopherol (Vit E, 400 IU/day) during 28 consecutive days before the second exercise testing. This supplementation dosage has been demonstrated to inhibit the release of IL-6 from skeletal muscle in humans (Fischer et al. 2004).

Before (presupplementation) and after (postsupplementation) vitamin supplementation, subjects performed a test consisting of 1.5 h running on a treadmill at the speed corresponding to the previously calculated 75% of the individual maximum oxygen consumption (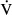 O_2max_). In earlier studies, this performance has resulted in elevated IL-6 and hepcidin serum levels (Peeling et al. 2009b). Venous blood samples from a catheter placed in the cubital vein were obtained at baseline, immediately posttrial (0 h), and at 3, 6, and 10 h posttrial, in order to analyze hepcidin, IL-6, C-reactive protein (CRP) and iron-related hematological parameters. On the day of the trials, subjects arrived to the laboratory at 6:30 a.m. Previous dinner and breakfast consisting of 200 g pasta with tomato sauce and milk with cereals, respectively, were standardized.

### Measurements

#### Maximum oxygen consumption

One week before the presupplementation test, subjects underwent an additional test to evaluate their individual 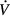
O_2max_. Details of the test and calculation of 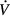
O_2max_ has been published elsewhere (Rabadan et al. 2011).

#### Vitamins C and E

Vitamin C was analyzed in plasma by reversed phase high-performance liquid chromatography (RP-HPLC) (Sykam Fürstenfeldbruck Germany) using UV-detection (UV-ViS 243 nm, Knauer, Berlin, Germany). Separation was carried out on an Inertsil RP-18 column (250 mm × 4.6 mm, Machery.Nagel, Düren, Germany) and an isocratic mobile phase (Steffan 1999). Vitamin E (*α*-tocopherol) was analyzed by reversed RP-HPLC (Sykam Fürstenfeldbruck Germany) in ethylene diamine tetraacetic acid (EDTA) plasma (Erhardt et al. 1999). The CV of these methods is 1.8% and 4.1% for vitamin C and E, respectively.

#### Interleukin-6

IL-6 was assessed by means of a commercially available ELISA kit (Quantikine HS; R&D Systems, Minneapolis, MN) following the instructions of the manufacturer. Samples were prepared in triplicate and the mean of three measurements was considered for later analysis. The CV of this method is 6.5% according to the manufacturer.

#### Hepcidin

Hepcidin serum levels were measured using the Hepcidin 25 bioactive competitive ELISA (DRG International, Marburg, Germany) following the manufacturer’s instructions. The CV of this method is 5.1%. The assay was previously shown to reliably detect decreased hepcidin levels under hypoxic conditions (Altamura et al. 2015).

#### Iron-related blood parameters

Transferrin saturation, serum iron, C-reactive protein (CRP), and haptoglobin were evaluated by standard and automated laboratory procedures (Synchron LX^® ^20 PRO, Beckman Coulter, Brea, CA). The CV was lower than 5% for all determinations.

### Statistical analysis

Results are expressed as mean and standard error of measurement (±SEM). Repeated measures analysis of variance (ANOVA) were used to analyze the results of each variable between pre and postsupplementation and among different blood sampling times (baseline and 0, 3, 6, and 10 h post trial). Post hoc Bonferroni was used to determine if specific trial differences existed.

Concentration of vitamins C and E were compared pre and postsupplementation by means of a paired *t*-test. The analyses were performed using SPSS 20.0 and the level of significance was set at *P *< 0.05.

## Results

### Serum concentration of vitamins C and E

The 28-days supplementation period with vitamins C and E was effective in increasing the corresponding serum concentration, as baseline values at the postsupplementation trial were significantly elevated (Vit C: 12.4 ± 1.0 vs. 16.1 ± 1.9 mg/L, *P* = 0.043; and Vit E: 9477.2 ± 1063.9 vs. 11329.7 ± 1484.3 IU, *P* = 0.003; pre and postsupplementation, respectively).

### Inflammation

**Figure 1:**
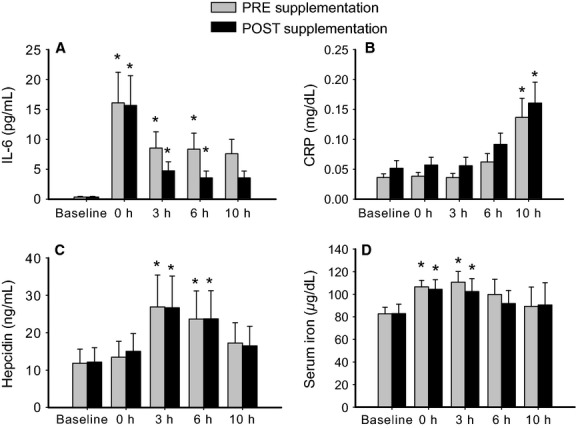
Exercise-induced response of IL-6 (A), CRP (B), hepcidin (C) and iron (D) in the serum before and after 28-days supplementation period with vitamins C and E. Error bars represent SEM. *designates significant differences (*P* < 0.05) compared to the corresponding baseline. No significant differences were found between pre and post vitamin supplementation.

Inflammation was assessed by measuring IL-6 and CRP serum levels. In the presence or absence of vitamin supplementation, IL-6 displayed a peak immediately after exercise and remained elevated for additional 6 h (Fig.1A). There was no statistical difference between vitamin supplementation or not, although IL-6 levels showed a tendency to decrease faster when vitamins were supplemented. Similarly, CRP levels were not altered by adding vitamins, and showed a progressive increase reaching a significant elevation compared to baseline values only 10 h after exercise (Fig.1B).

### Hepcidin and iron-related parameters

Compared to baseline, hepcidin levels showed a significant increase 3 h and 6 h post exercise in both, pre and postsupplementation conditions. But once again no differences were detected between supplementation or not (Fig.1C). Of note, IL-6 increase from baseline to 0 h post exercise did not show a relationship with peak hepcidin values (3 h post exercise) in any of the trials as tested using Pearson correlation coefficient (data not shown).

Similarly, serum iron levels (Fig.1D) displayed a significant increase immediately and 3 h post exercise compared to baseline that did not correlate with hepcidin levels in individual athletes. Importantly, serum iron levels remained unaffected by vitamin supplementation at any time point.

Transferrin saturation and haptoglobin levels did not differ either between pre and postsupplementation trials or compared to baseline at any time point (data not shown).

## Discussion

Iron deficiency among endurance athletes has been associated with postexercise elevation of IL-6 and a subsequent hepcidin up-regulation (Peeling et al. 2009a; Peeling 2010; Peeling et al. 2014). Because hepcidin decreases dietary iron uptake and iron release from iron recycling macrophages this may contribute to the iron deficiency observed in endurance athletes. Here we show that serum hepcidin significantly increased up to 6 h after exercise and fails to correlate with elevated levels of the circulating inflammatory cytokine IL-6 or serum iron levels. We further show that the release of IL-6 as well as serum iron and hepcidin levels are not significantly affected by the moderate supplementation with vitamins C and E during 28 consecutive days. Despite the fact that IL-6 tends to decrease faster after vitamin supplementation, hepcidin levels did not reflect a vitamin-dependent alteration. Overall, these results suggest that, rather than only IL-6, a combination of inflammatory and iron signals may contribute to postexercise hepcidin elevation and that this response is not affected by the chosen vitamin supplementation.

An immediate postexercise IL-6 peak followed by increasing hepcidin levels occurring within –6 h has been reported (Peeling et al. 2009b). Our study confirms the previously reported time course for both parameters under similar experimental conditions. However, elevation of IL-6 and CRP in the recovery phase (see 6 h in Fig.1A and 10 h in Fig.1B) was accompanied by decreasing hepcidin levels (Fig.1C). This lack of correlation may be explained by the subjects’ iron status. As such, Peeling and coworkers have recently performed an experiment to elucidate the impact of iron status on exercise-induce hepcidin serum levels (Peeling et al. 2014). Despite observing similar postexercise levels of IL-6 and serum iron as in this study, hepcidin elevation after running was described to be dependent on the initial iron status of their subjects. In our study, the group of athletic subjects was homogeneous in terms of iron status and presented normal levels of serum ferritin (>50 ug/L). These data suggest that iron-dependent signaling due to elevated serum iron levels as well as mild inflammatory IL6-dependant signals may be responsible for the increase in hepcidin levels in response to exercise. Finally, other factors such as the newly described erythroferrone (Kautz et al. 2014a,b) may contribute to regulate iron metabolism after exercise. Notably, erythroferrone is identical to an also recently discovered myokine termed myonectin or CTRP15 (Seldin et al. 2012). If whole body exercise leads to a release of CTRP15 and contributes to the regulation of iron metabolism remains to be investigated (reviewed in Gassmann and Muckenthaler 2015).

Some studies have shown a marked reduction of IL-6 release upon carbohydrate ingestion during cycling (Starkie et al. 2001; Febbraio et al. 2003). Sim and colleagues attempted to inhibit exercise-induced IL-6 release using carbohydrate loading during endurance running and observed that serum iron and IL-6 were significantly elevated immediately postrun in both, carbohydrate and placebo groups (Sim et al. 2012). In addition, serum hepcidin concentration recorded 3 h post run in the presence or absence of loaded carbohydrates was significantly elevated. Similar to our results, that study did not show a significant supplementation-dependent decrease in IL-6 compared to the placebo trial. On the other hand, vitamin dosage in our study may have been too low taking into account that our experiment involved whole body exercise (e.g., running 1.5 h at 75% 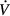
O_2max_) as compared to the Fischer study (Fischer et al. 2004) where a two-leg exercise mode was used (knee extension 3 h at 50% 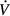
O_2max_). In line with our results a previous study showed that 14-days supplementation period with vitamins C and did not prevent postexercise elevation of IL-6 during running (Petersen et al. 2001), suggesting that the exercise mode (impact during running vs. no impact during cycling) may play an important role in IL-6 release during exercise.

In summary, moderate supplementation with vitamin C and vitamin E failed to significantly inhibit both, the release of IL-6 and hepcidin. At present we cannot elucidate the relative contribution of the iron status and IL-6 levels to postexercise hepcidin elevation as we were unable to significantly reduce the IL-6 levels. However, the tendency on faster IL-6 reduction upon vitamin supplementation may suggest that iron status, rather than inflammation alone, also contributes to the hepcidin response observed after exercise. Of note we standardized the diet and the time of the experiments, thereby controlling for any potential effect on hepcidin expression due to nutritional status or circadian rhythm.

Finally, our work has practical applications in line with those already suggested (Peeling et al. 2009b; Peeling 2010; Peeling et al. 2014): Vitamin supplementation in healthy and well-trained athletes has come into fashion. It is argued that vitamin supplementation blunts the inflammatory response often observed after exercise. At least, with respect to hepcidin and iron levels, this study reveals no immediate correlation. This suggests that the use of vitamin C and E for controlling iron metabolism had no effect at least at the chosen dosage. However, if iron supplementation is prescribed to athletes, physicians, and other professionals should take into account the timing of iron administration, as a high reproducible peak in hepcidin is observed 3–6 h after endurance running.
